# Self-Rated Effectiveness of Microdosing With Psychedelics for Mental and Physical Health Problems Among Microdosers

**DOI:** 10.3389/fpsyt.2019.00672

**Published:** 2019-09-13

**Authors:** Nadia R. P. W. Hutten, Natasha L. Mason, Patrick C. Dolder, Kim P. C. Kuypers

**Affiliations:** Department of Neuropsychology & Psychopharmacology, Faculty of Psychology & Neuroscience, Maastricht University, Maastricht, Netherlands

**Keywords:** psychedelics, microdosing, self-medication, symptom alleviation, efficacy, psilocybin, LSD

## Abstract

**Background:** There is a growing interest in the use of psychedelic substances for health related purposes, including symptom relief for disorders like anxiety, depression, and pain. Although the focus of recent clinical trials has been on high doses of these substances, anecdotal evidence suggests that low (micro) doses are also effective, and may be more suitable for certain conditions. Nonetheless, empirical evidence regarding the efficacy of microdosing with psychedelics for symptomatic relief is lacking. The present study aimed to investigate, by means of an online questionnaire, the self-rated effectiveness (SRE) of microdosing with psychedelics (MDP) for mental and physiological disorders compared to the conventional prescribed treatment and to regular doses of psychedelics.

**Methods:** An online questionnaire was launched on several websites and fora between March and July 2018. Respondents who had consented, were 18 years of age or older, had experience with microdosing and were diagnosed with at least one mental or physiological disorder by a medical doctor or therapist (*N* = 410; 7.2%) were included in the analyses. Odds ratio were calculated to compare the SRE of MDP with conventional treatment, and regular psychedelic doses for mental and physiological diagnoses for each of the three effectiveness questions (“Did it work,” “Symptom disappear,” “Quality of life improved”).

**Results:** Odds ratio showed that SRE of MDP was significantly higher compared to that of conventional treatments for both mental and physiological diagnoses; and that these effects were specific for ADHD/ADD and anxiety disorders. In contrast, SRE of MDP was lower compared to that of higher, regular psychedelic doses for mental disorders such as anxiety and depression, while for physiological disorders no difference was shown.

**Conclusion:** This study demonstrates that SRE of MDP to alleviate symptoms of a range of mental or physiological diagnoses is higher compared to conventionally offered treatment options, and lower than regular (‘full’) psychedelic doses. Future RCTs in patient populations should objectively assess the effectivity claims of psychedelics, and whether these are dose related, disorder specific, and superior to conventional treatments.

## Introduction

As of the last few years, there has been an increasing visibility and interest in the use of low doses of psychedelics, such as lysergic acid diethylamide (LSD) and psilocybin, for beneficial health-related purposes. Referred to as “microdosing,” users report consuming about one tenth of a recreational dose (1, 2), to enhance daily functions, without inducing a profound altered state of consciousness (2–9). While the primary motivation to microdose is indeed to enhance performance, including creativity and mental concentration (10), it is also reported to be used to alleviate psychological and physical symptoms, such as anxiety and headache (10–12). However, empirical evidence regarding the efficacy of microdosing with psychedelics to relieve the aforementioned symptoms is currently lacking.

More extensive evidence on the potential therapeutic value of psychedelic substances has been shown after use of regular (larger) doses which induce typical full effects and a profound altered state of consciousness. Recent clinical studies have suggested that LSD (13), psilocybin (14), ayahuasca (15), and methylenedioxymethamphetamine (MDMA) (16, 17), in combination with psychological support, can provide therapeutic relief for those suffering from post-traumatic stress disorder (PTSD), anxiety, and depression. Additionally, earlier studies demonstrated that psychedelics also provided physical symptom relief, for example in patients with pathologic pain (18). Nonetheless, a psychedelic experience, characterized by acute alterations in perception and cognition, and amplified emotional states (19), may not always be necessary in case of the latter, or not preferable based on individuals’ (personality) traits (20) or previous (in)experience with psychedelic substances (21). Furthermore, although physically safe, psychedelic experiences can prove challenging and thus psychological support is encouraged during and after the experience. Taken together, a recreational, full dose can prove costly and impractical for certain disorders, requiring individuals to be supervised in a controlled, clinical environment.

Overall, anecdotal reports and small clinical trials support the potential therapeutic utility of psychedelic substances in reducing symptomatology of a range of mental and physiological disorders. However, it has yet to be shown whether a psychedelic experience as induced by a “full” regular dose is necessary to produce symptom relief, or whether (repeated) sub-perceptual doses have therapeutic potential as well. The present study aimed to investigate, by means of an online questionnaire, the self-rated effectiveness (SRE) of microdosing with psychedelics (MDP) for mental and physiological disorders compared to the conventional prescribed treatment and to regular doses of psychedelics.

## Methods

### Design

An online questionnaire was advertised to psychedelic users on several psychedelic websites and fora between March and July 2018. The questionnaire was not explicitly targeted to microdosers, and ‘microdosing’ was not mentioned in the advert in order to obtain a rate of base rate of microdosing in the psychedelic user groups. To be eligible to fill out the survey, respondents had to be ≥18 years and have experience with a psychedelic substance. After having read the study information and having had the opportunity to ask questions about the study, respondents gave their informed consent in order to continue with the survey. Ethics approval was received from the Ethics Review Committee of Psychology and Neuroscience (ERCPN-177_06_03_2017). Qualtrics was used as the platform to create the survey.

### Questionnaire

#### Demographic Information

Demographic details included age, gender, continent of origin, daily occupation, and the highest level of education. Daily occupation consisted of six pre-set options that respondents could choose from; learning/studying, physical work, computer/office work, working with people, travelling, and creative work. The level of education consisted of three pre-set categories; primary (e.g. elementary school), secondary (e.g. high school, academies, gymnasium) and tertiary education (e.g. university, trade school, college).

#### Psychedelic Substance Use History

Respondents were asked whether they have had experience with LSD, 1P-LSD, ALD-52/1A-LSD, psilocybin (including psilocybin-containing truffles or mushrooms), ayahuasca, DMT, 5-MeO-DMT, Salvinorin A, Mescaline, MDMA/Ecstasy, NBOMe’s, 2C’s, or any other psychedelic drug in either a microdose, and/or regular dose, which was defined as having “a full psychedelic experience.” Further questions about motivations and side effects of microdosing, as well as the microdosing schedule used are reported elsewhere (10).

#### Mental and Physiological Diagnoses

Respondents were asked whether a medical doctor or therapist diagnosed them with a psychiatric, neurological, or physical disorder. When affirmed, they were asked which of the pre-set disorders applied: depression, anxiety/panic disorder, attention deficit hyperactivity disorder (ADHD) or attention deficit disorder (ADD), bipolar disorder, schizophrenia, obsessive compulsive disorder (OCD), autism/Asperger syndrome, antisocial behavior disorder, borderline personality disorder, substance abuse disorder, Tourette’s, Parkinson’s, epilepsy, migraine, cluster headache, multiple sclerosis (MS), and/or chronic pain. Furthermore, they had the option to enter free text in a text box when the disorder was not listed.

Disorders were clustered afterwards into main categories according to the classification system of the two leading diagnostic manuals, the DSM-5 for mental disorders and the ICD-10 for physiological disorders which resulted in 14 sub-categories for mental disorders and 11 sub-categories for physiological disorders (**Table 1**). When free text was entered, the response was manually re-classified in the best matching category.

**Table 1:** Number (percentage) of diagnoses per sub-category of mental and physiological disorders, further separated into those who received conventional treatment and those who used psychedelics to self-medicate.

Diagnoses	Number (%) of respondents who are diagnosed	Number (%) diagnoses per category	Number (%) of diagnoses that received conventional treatment	Self-medication with a psychedelic substance		
				Number (%) that only microdosed	Number (%) that only used regular dose	Number (%) that used both, microdose and regular dose
**Mental disorders (DSM-5 categories**)						
Neurodevelopmental disorders	ADHD/ADD 153 (37.3), Autism/Asperger 32 (7.8), Tourette 3 (0.7)	188 (45.6)	140 (74.5)	20 (10.6)	5 (2.7)	55 (29.3)
Schizophrenia spectrum and other psychotic disorders	Schizophrenia 12 (2.9)	12 (2.9)	9 (75.0)	–	2 (16.7)	3(25.0)
Bipolar and related disorders	Bipolar 37 (9.0)	37 (9.0)	28 (75.7)	–	2 (5.4)	10 (27.0)
Depressive disorders	Depression 298 (72.7), PMMD 1 (0.2)	299 (72.9)	260 (87.0)	17 (5.7)	22 (7.4)	206 (68.9)
Anxiety disorders	Anxiety/panic disorders 228 (55.6)	228 (55.6)	181 (79.4)	14 (6.1)	9 (3.9)	92 (40.4)
OCD and related disorders	OCD 27 (6.6)	27 (6.6)	18 (66.7)	2 (7.4)	1 (3.7)	9 (33.3)
Trauma- and stressor-related disorders	PTSD 19 (4.6)	19 (4.6)	15 (78.9)	2 (10.5)	3 (15.8)	6 (31.6)
Feeding and eating disorder	Eating disorder^b^ 4 (1.0)	4 (1.0)	3 (75.0)	–	1 (25.0)	1 (25.0)
Sleep-wake disorder	Sleep-wake disorders^a^ 4 (1.0)	4 (1.0)	2 (50.0)	–	–	–
Sexual dysfunctions	Erectile dysfunction 1 (0.2)	1 (0.2)	1 (100.0)	–	–	1 (100.0)
Gender Dysphoria	Gender dysphoria 2 (0.5)	2 (0.5)	2 (100.0)	–	1 (50.0)	1 (50.0)
Disruptive, impulse control and conduct disorders	BFRB 1 (0.2)	1 (0.2)	1 (100.0)	–	–	1 (100.0)
Substance-related and addictive disorders	Substance abuse disorder 49 (12.0)	49 (12.0)	30 (61.2)	–	2 (4.1)	19 (38.8)
Personality disorders	Antisocial 10 (2.4), Borderline 20 (4.9), Schizoid personality disorder 1 (0.2), dependent personality disorder 1 (0.2)	32 (7.8)	24 (75.0)	1 (3.1)	3 (9.4)	9 (28.1)
**Physiological disorders (ICD-10 categories)**						
II C00-D48 Neoplasms	Cancer 2 (0.5), Hodgkin’s lymphoma 1 (0.2)	3 (0.7)	2 (66.7)	–	–	–
IV E00-E90 Endocrine, nutritional and metabolic diseases	Hashimoto’s thyroiditis 1 (0.2)	1 (0.2)	1 (100.0)	–	–	–
V F00-F99 Mental and behavioral disorders	Post-concussive syndrome 1 (0.2)	1 (0.2)	–	–	–	–
VI G00-G99 Diseases of the nervous system	Epilepsy 12 (2.9), Parkinson 2 (0.5), MS 1 (0.2), Cluster headaches 15 (3.7), Migraines 60 (14.6), Chronic pain 56 (13.7), Daily persistent headache 1 (0.2), Dystonia 1 (0.2)	148 (36.1)	96 (64.9)	7 (4.7)	10 (6.8)	30 (20.3)
VII H60-H95 Diseases of the ear and mastoid process	Almost deaf 1 (0.2)	1 (0.2)	1 (100.0)	–	–	–
XI K00-K93 Diseases of the digestive system	Crohns disease 2 (0.5), IBS 2 (0.5)	4 (1.0)	2 (50.0)	–	–	1 (25.0)
XII L00-L99 Diseases of the skin and subcutaneous tissue	Lupus erythematosus 1 (0.2)	1 (0.2)	1 (100.0)	–	–	1 (100.0)
XIII M00-M99 Diseases of the musculoskeletal system and connective tissue	Fibromyalgia 2 (0.5)	2 (0.5)	2 (100.0)	–	–	1 (50.0)
XVII Q00-Q99 Congenital malformations, deformations and chromosomal abnormalities	Ehlers Danlos Syndrome 1 (0.2)	1 (0.2)	1 (100.0)	–	–	–
XIX S00-T98 Injury, poisoning and certain other consequences of external causes	Food sensitivities 1 (0.2)	1 (0.2)	1 (100.0)	–	–	1 (100.0)
Non-classified disorders	Daytime sleepiness 1 (0.2)	1 (0.2)	1 (100.0)	–	–	–

#### Effectiveness of Conventional Prescribed Treatment

When respondents indicated to have been diagnosed with a specific mental or physiological disorder, they were asked whether they were offered treatment for that particular disorder. In case answers were affirmative (medication, therapy, or both) these questions were followed by three extra questions about treatment efficacy which could be answered negative (“definitely not,” “probably not”) or positive (“probably yes,” “definitely yes”). The questions were “Do you feel the treatment worked,” “Did the symptoms disappear to an extent at which daily functioning was not compromised any longer,” and “Did your quality of life improve.”

#### Effectiveness of Psychedelic Self-Medication

Respondents were asked whether they have used a psychedelic in order to treat their diagnosed disorder. When affirmative, this was followed by a question which psychedelic substance they used to alleviate the symptoms of the particular disorder, with pre-set options: LSD, 1P-LSD, ALD-52/1A-LSD, psilocybin, ayahuasca, DMT, 5-MeO-DMT, Salvinorin A, Mescaline, MDMA/Ecstasy, NBOMe’s, 2C’s or any other psychedelic substance. Followed by the question whether they used the substance in a microdose, a regular dose, or both. Additionally, the same three questions about treatment efficacy — as those asked for conventional treatment — were asked per psychedelic substance and dosing (‘micro’ and/or regular).

### Statistical Analysis

Data entered the statistical program SPSS (version 24.0). Respondents who did not give their consent, were not 18 year or older, did not complete the questionnaire, did not have microdosing experience, and did not have any mental and/or physiological diagnosis were excluded (*N* = 5,271) from the analyses. Frequencies are reported for gender, education, continent of origin, daily occupation, and psychedelic drug use history. Mean ( ± *SD*) is given for age.

Frequencies are reported for the total number of mental and physiological diagnoses in general, and more specific, per sub-category, for conventional treatment, the use of self-medication with a microdose, a regular dose, and both. The most frequently used psychedelics for self-medication are also reported.

To compare the effectiveness of self-medication with psychedelic microdoses with conventional treatment, and regular psychedelic doses, binary logistic regressions were conducted for the mental (total) and physiological (total) diagnoses for each of the three effectiveness questions. This resulted in odds ratio (OR) values for the three questions. In case of significant results, separate binary logic regressions were conducted for each category within the mental or physiological diagnosed group in order to examine whether this effect was disorder-specific.

Even though ADHD/ADD and autism/Asperger’s are both placed in the same category of ‘neurodevelopmental disorders’ in the DSM-5, the core symptoms of both disorders are different. The scope symptoms of ADHD/ADD are defined as having impairments in attention, impulse control, and hyperactivity; while symptoms of autism/Asperger’s are defined as having deficits in social communication and interaction, and restricted repetitive behavior (22–24). Therefore, *ad hoc* analyses have been conducted in order to examine whether ADHD/ADD and/or autism/Asperger’s account for the results of neurodevelopmental disorders. When cell count was less than 10 events per independent variable (EVP), no regression was conducted (26). For each OR, 95% confidence intervals (CIs) are given and statistical significance was set at *p* = 0.05. An OR of 1.5 is defined as small, as medium, and 3 as large (Sullivan and Feinn, 2012).

## Results

### Demographic Information

In total, 3,590 out of 5,681 respondents consented, were 18 years or older, and completed the questionnaire. It took respondents about 16 min to complete the questionnaire, depending on the number of psychedelic substances a person had ever used before, whether they microdosed and whether they were diagnosed with a disorder. One third (*N* = 1,116; 31.1%) of the respondents indicated to have microdosed with at least one psychedelic substance. More than one-third (*N* = 410; 36.7%) of the microdosers indicated to have been diagnosed with at least one mental or physiological disorder by a medical doctor or therapist, the remaining 1,414 respondents who did not microdose and/or were not diagnosed with a disorder were removed from further analyses. Group demographics and detailed drug use history for the whole sample are presented separately, see Hutten et al. (10).

Respondents’ mean ( ± *SD*) age was 28.9 (± 10.1) years with a maximum age of 72 (*N* = 1); 306 (74.6%) were males aged on average 29.1 (± 10.4) years, 94 (22.9%) females aged on average 28.8 (± 9.3) years, and 10 (2.4%) classified themselves as “other” and had an average age of 25.1 (± 5.9) years. Most of them attended tertiary education (*N* = 290; 70.7%), the prevailing daily occupation was learning/studying (*N* = 124; 30.2%), and the majority of our sample originated from North-America (*N* = 276; 67.3%).

The highest level of education for the other one third of the sample was primary (*N* = 6; 1.5%) and secondary (*N* = 114; 27.8%). Other continents of origin were Europe (*N* = 103; 25.1%), Australia (*N* = 16; 3.9%), Asia (*N* = 6; 1.5%), South-America (*N* = 5; 1.2%) and Africa (*N* = 4; 1.0%), and daily occupation of the others in the sample consisted of computer/office work (*N* = 99; 24.1%), working with people (*N* = 65; 15.9%), physical work (*N* = 53; 12.9%), creative work (*N* = 59; 14.4%), and travelling (*N* = 3; 0.7%); 1.7% (*N* = 7) did not answer this question.

All microdosers reported to have had experience with regular doses of psychedelics, of which psilocybin (*N* = 355; 86.6%), LSD (*N* = 325; 79.3%), and MDMA/ecstasy (*N* = 263; 64.1%) were the most frequently reported. The most frequently reported psychedelics for microdosing were psilocybin (*N* = 248; 60.5%), LSD (*N* = 231; 56.3%), and 1P-LSD (*N* = 43; 10.5%).

### Mental and Physiological Diagnoses

In total, there were 901 mental diagnoses and 161 physiological diagnoses reported. This total number (1,062) is higher than the included sample (*N* = 410) of microdosers because the majority (*N* = 298; 72.7%) indicated to be diagnosed with more than one disorder. The average number of diagnoses among the respondents was 2.5 diagnoses. A minority (*N* = 9; 2.2%) did not disclose the exact disorder they were diagnosed with.

The three most prevalent mental diagnosed disorders in descending order are depressive disorders (*N* = 298; 72.7%), anxiety disorders (*N* = 228; 55.6%), and ADHD/ADD (*N* = 153; 37.3%). The three most prevalent physiological diagnosed disorders are migraines (*N* = 60; 14.6%), chronic pain (*N* = 56; 13.7%), and cluster headaches (*N* = 15; 3.7%). The number of diagnoses per sub-category are presented in **Table 1**.

## Treatment

The majority of mental diagnoses [number of diagnoses (*N*) = 714; percentage (%) = 79.2] were prescribed conventional (non)pharmacological treatments. Psychedelics were used to self-medicate in more than half of the mental diagnoses (*N* = 520; 57.7%) of which the majority refers to both a regular dose and microdose (*N* = 413; 79.4%). In the other one-fifth of mental diagnoses only a microdose (*N* = 56; 10.8%) or only a regular dose (*N* = 51; 9.8%) was used to self-medicate.

The most reported psychedelics used to self-medicate for mental disorders in descending order are: psilocybin (*N* = 297; 57.1%), LSD (*N* = 248; 47.7%), and 1P-LSD (*N* = 68; 13.1%) in microdoses, and psilocybin (*N* = 336; 64.6%), LSD (*N* = 264; 50.8%), and MDMA (*N* = 115; 22.1%) in regular doses.

The majority of physiological disorders (*N* = 123; 76.4%) were treated with conventional therapy. In one-third (*N* = 51; 31.7%) of the cases psychedelics were used to self-medicate of which the majority was both with a microdose and a regular dose (*N* = 34; 66.7%); the remainder self-treated with only a microdose (*N* = 7; 13.7%) or a regular dose (*N* = 10; 19.6%). The most reported psychedelics used in order to self-medicate for these phsychological disorders in descending order are: psilocybin (*N* = 28; 54.9%), LSD (*N* = 14; 27.5%), and DMT (*N* = 3; 5.9%) in microdoses; and psilocybin (*N* = 30; 58.8%), LSD (*N* = 20; 39.2%), and MDMA (*N* = 4; 7.8%) for regular doses.

Details of the treatment sub-categories are presented in **Table 1**.

### Effectiveness of Psychedelic Microdosing Compared to Conventional Treatment

**Figure 1:**
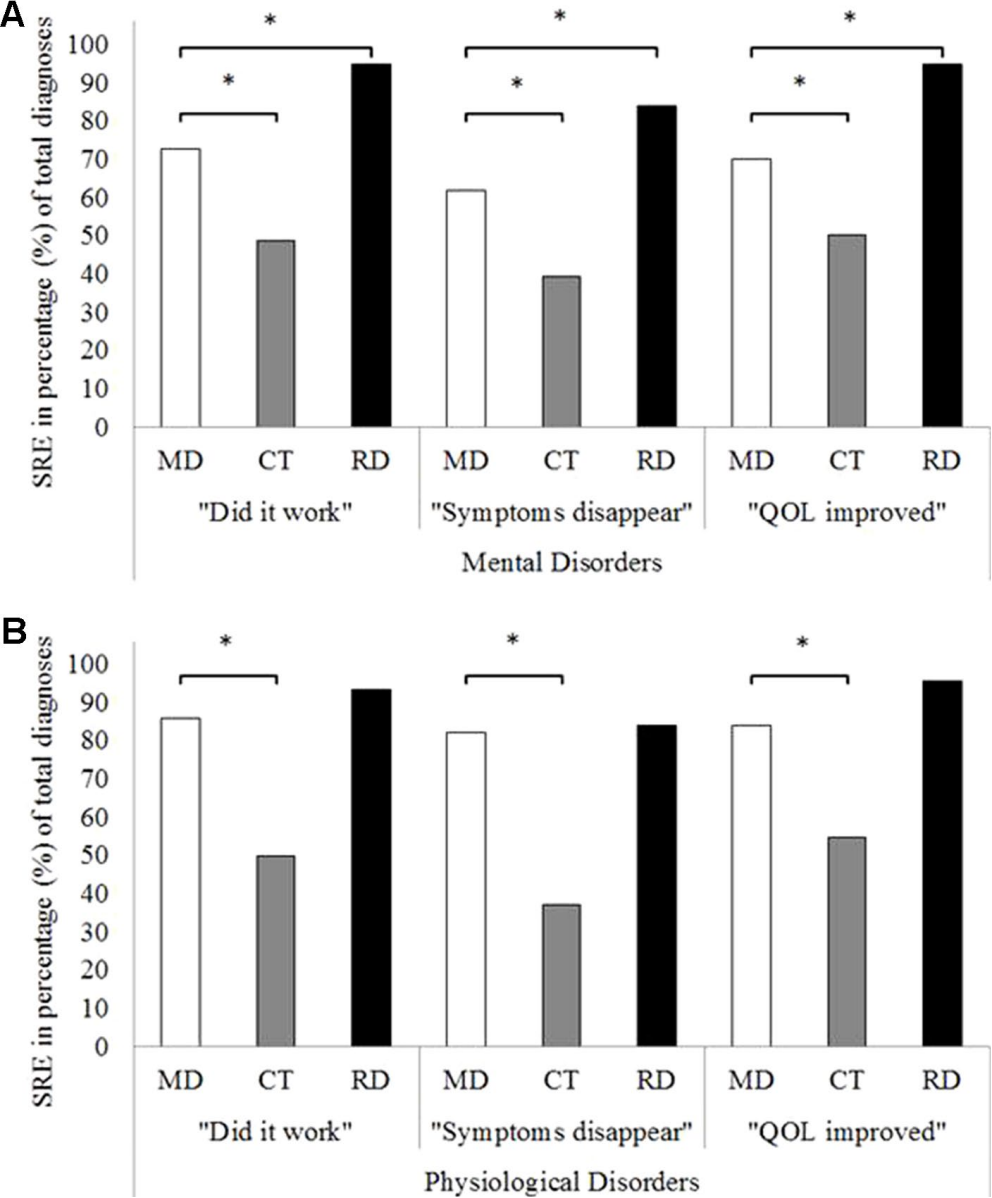
Overall self-rated effectiveness of psychedelic microdoses, conventional treatment, and regular doses of a psychedelic on the three effectiveness questions for mental disorders **(A)** and for physiological disorders **(B)**. *Signifies statistically significant binary logistic regression *p* < 0.05. SRE, self-rated effectiveness; MD, microdose; CT, conventional treatment; RD, regular dose.

Binary logistic regression analysis demonstrated that SRE of MDP to treat mental disorders was rated significantly higher compared to that of the conventional, prescribed treatment as indicated by statistically significant OR for the three questions OR (did it work) = 2.77 (*p* < 0.01; 95% CI [2.19, 3.50]); OR (symptoms disappear) = 2.48 (*p* < 0.01; 95% CI [1.97, 3.10]), OR “QOL improved” = 2.30 (*p* < 0.01; 95% CI [1.82, 2.90]) (**Figure 1A**).

Separate binary logistic regressions per mental sub-category showed that MDP was only more effective than conventional therapy for neurodevelopmental and anxiety disorders. Ad hoc analyses of neurodevelopmental disorders revealed that the MDP was rated more effective than conventional therapy for diagnoses of ADHD/ADD, while there were no significant results in the autism/Asperger’s category. For the other listed mental disorders statistical significance was either not proven for all three questions (**Table 2**) or it was not possible to calculate due to the low cell count (this was the case for six sub-categories: e.g. trauma- and stressor-related disorders; feeding and eating disorders; sleep-wake disorders; sexual dysfunctions; gender dysphoria; and disruptive, impulse control and conduct disorders).

**Table 2:** The odds ratio for SRE of MDP compared to conventional treatment and regular doses compared for each of the three effectiveness questions per sub-category of mental disorders^a^.

	“Did it work?”		“Symptoms disappeared?”		“QOL improved?”	
	OR (95% CI)	*P*-value	OR (95% CI)	*P*-value	OR (95% CI)	*P*-value
**Microdose compared to conventional therapy**						
Neurodevelopmental disorders ADHD/ADD autism/asperger	4.33 (2.06, 9.12) 11.66 (3.46, 39.34) 6.25 (0.64, 60.94)	<0.01 <0.01 0.12	2.56 (1.47, 4.46) 3.40 (1.77, 6.52) 2.67 (0.61, 11.70)	<0.01 <0.01 0.19	3.63 (1.87, 8.05) 8.62 (3.23, 22.98) 1.81 (0.35, 9.24)	<0.01 <0.01 0.48
Bipolar and related disorders	4.62 (1.06, 20.01)	0.04	4.00 (0.92, 17.33)	0.06	3.47 (0.80, 15.03)	0.10
Depressive disorders	1.89 (1.34, 2.66)	<0.01	1.87 (1.32, 2.64)	<0.01	1.35 (0.96, 1.89)	0.09
Anxiety disorders	6.06 (3.50, 10.44)	<0.01	4.59 (2.78, 7.59)	<0.01	5.78 (3.35, 9.98)	<0.01
OCD and related disorders	3.33 (0.68, 16.29)	0.14	4.16 (0.91, 19.03)	0.07	1.43 (0.32, 6.49)	0.64
Substance-related and addictive disorders	3.94 (1.07, 14.44)	0.04	1.34 (0.43, 3.14)	0.61	5.54 (1.35, 22.77)	0.02
Personality disorders	5.91 (1.06, 32.92)	0.04	5.00 (1.07, 23.46)	0.04	7.86 (0.87, 71.06)	0.07
**Microdose compared to regular dose**						
Neurodevelopmental disorders ADHD/ADD autism/asperger	1.30 (0.48, 3.52) 5.33 (1.32, 21.52) <0.01 (0.00, 0.00)	0.61 0.02 0.99	1.53 (0.76, 3.05) 2.24 (0.97, 5.14) 1.36 (0.29, 6.42)	0.23 0.06 0.70	0.52 (0.18, 1.53) 1.77 (0.48, 6.53) <0.01 (0.00, 0.00)	0.23 0.40 0.99
Bipolar and related disorders	0.40 (0.36, 4.47)	0.46	0.40 (0.04, 4.47)	0.46	0.40 (0.04, 4.47)	0.46
Depressive disorders	0.03 (0.01, 0.08)	<0.01	0.14 (0.09, 0.22)	<0.01	0.04 (0.02, 0.09)	<0.01
Anxiety disorders	0.15 (0.04, 0.50)	<0.01	0.26 (0.12, 0.60)	<0.01	0.25 (0.09, 0.68)	<0.01
OCD and related disorders	0.83 (0.11, 6.26)	0.86	0.69 (0.12, 3.96)	0.67	0.56 (0.08, 3.94)	0.56
Substance-related and addictive disorders	0.75 (0.15, 3.84)	0.73	0.29 (0.07, 1.31)	0.11	0.32 (0.03, 3.32)	0.34
Personality disorders	0.50 (0.04, 6.44)	0.60	0.67 (0.09, 4.99)	0.69	1.10 (0.06, 20.01)	0.95

QOL, quality of life; OR, Odds Ratio; CI, confidence interval; ADHD/ADD, attention deficit hyperactivity disorder/attention deficit disorder; OCD, obsessive-compulsive disorder.

a Diagnoses with cell count less than 10 are not reported.

In addition, binary logistic regression analysis demonstrated that SRE of MDP to treat physiological disorders was significantly higher compared to that of conventional treatment as indicated by statistically significant OR for the three questions OR “did it work” = 6.14 (*p* < 0.01; 95% CI [2.54, 14.86]); OR “symptoms disappear” = 7.74 (*p* < 0.01; 95% CI [3.41, 17.59]); and OR “QOL improved” = 4.36 (*p* < 0.01; 95% CI [1.87, 10.16]), **Figure 1B**).

A separate binary logistic regression for the sub-category ‘diseases of the nervous system’ of the physiological disorders revealed that MDP was rated to be more effective compared to conventional treatment as indicated by statistically significant OR for the three questions OR “did it work” = 6.78 (*p* < 0.01; 95% CI [2.63, 17.49]); OR “symptoms disappear” = 7.71 (*p* < 0.01; 95% CI [3.23, 18.38]); and OR “QOL improved” = 4.59 (*p* < 0.01; 95% CI [1.87, 11.31]). For all other listed physiological disorders binary logistic regression was not possible to calculate due to the low cell count.

### Effectiveness of Psychedelic Microdoses Compared to Regular Doses

Binary logistic regression analysis demonstrated that MDP was rated as less beneficial compared to regular doses for mental disorders as indicated by statistically significant OR for the three questions (OR “did it work” = 0.15 (*p* < 0.01; 95% CI [0.10, 0.24]); OR “symptoms disappear” = 0.31 (*p* < 0.01; 95% CI [0.23, 0.42]); and OR “QOL improved” = 0.13 (*p* < 0.01; 95% CI [0.08, 0.21], **Figure 1A**). However, separate binary logistic regressions per sub-category showed that self-medication with microdoses were statistically less efficacious than regular psychedelic doses for depressive and anxiety disorders on all three effectiveness questions (see **Table 2**) or it was not possible to calculate due to the low cell count (this was the case for six sub-categories: e.g. trauma- and stressor-related disorders; feeding and eating disorders; sleep-wake disorders; sexual dysfunctions; gender dysphoria; and disruptive, impulse control and conduct disorders).

Binary logistic regression analysis also demonstrated that there was no difference in SRE when comparing microdoses and regular doses to treat physiological disorders as indicated by statistically non-significant OR for the three questions (OR “did it work” = 0.45 (*p* = 0.27; 95% CI [0.12, 1.86]); OR “symptoms disappear” = 0.86 (*p* = 0.79; 95% CI [0.29, 2.55]); and OR “QOL improved” = 0.25 (*p* = 0.09; 95% CI [0.05, 1.25]; **Figure 1B**).

## Discussion

The present study aimed to investigate, by means of an online questionnaire, the self-rated effectiveness (SRE) of self-medication with psychedelic microdoses for diagnosed mental and physiological disorders, compared to conventional treatments and regular doses of psychedelics. Overall, findings showed that SRE of MDP on all three effectiveness questions (“Did it work?”, “Did symptoms disappear?”, “Did your quality of life improve?”) was higher compared to that of conventional treatments for both mental and physiological diagnoses. In contrast, SRE of microdoses was lower compared to that of regular psychedelic doses for mental disorders, while for physiological disorders no difference was shown. Of note, the aforementioned effects were shown to be disorder specific. Specifically, compared to conventional treatments, further analysis demonstrated that MDP was only rated more beneficial on all three effectiveness questions for neurodevelopmental and anxiety disorders, *ad hoc* analyses revealed that only ADHD/ADD accounted for the results for neurodevelopmental disorders. Whereas compared to regular doses of psychedelics, MDP was rated to be less beneficial on all three effectiveness questions only for depression and anxiety.

The current survey demonstrates that self-medication with MDP was experienced to be more effective compared to conventional treatment in case of anxiety, ADHD/ADD, and physiological disorders such as pain. These findings are in line with anecdotal reports and interview studies reporting the use of psychedelic microdoses to substitute conventional prescribed medications (11, 12, 26). As no experimental comparison between MDP and conventional (non)pharmacological treatments for disorders exists, one can only speculate about the reasons why MDP is found to be more effective. First, MDP produces potentially less unwanted effects compared to conventional pharmacological treatments. For instance, users reported that their traditional stimulants for ADHD cause a crash after use while MDP did not (26). Additionally, compared to traditionally offered medications which are taken daily or even several times a day, microdosers do not usually consume the substance daily (2, 10), thus reducing potential costs and side effects, and even potentially reducing the number of reminders to the patient of being ill.

Although the three effectiveness questions (‘worked’, ‘disappeared’, ‘QOL’) were only statistically significant when MDP was used for anxiety, ADHD/ADD and physiological disorders, other disorders such as depressive, bipolar, substance-related and personality disorders were rated as effective on some of the questions, e.g., depression (‘worked’ and ‘disappeared’), bipolar (‘worked’), substance-related (‘worked’ and ‘QOL’), and personality (‘worked’ and ‘disappear’). Interestingly, OCD was not rated to be more effective on any of the questions compared to conventional treatments, and while anecdotal evidence is inconclusive about the effects of MDP on OCD (27), this might indicate that MDP is not effective in treating OCD. In order to understand these differences in SRE of MDP for different disorders, RCTs are needed to objectively examine the reported effects as well as the underlying mechanisms. This knowledge is necessary in the case that psychedelics are approved for therapeutic use for specified indications like PTSD (MDMA) and depression (psilocybin), and off-label prescriptions are being considered.

When comparing SRE of MDP and regular doses of psychedelics, it was found that microdoses were rated to be less effective than regular doses when self-medicating for depression and anxiety, whereas no difference was found for other disorders such as neurodevelopmental disorders, OCD related or physiological disorders such as chronic pain. The finding that only these two disorders were ‘dose-specific’ is interesting in light of recent clinical studies. Specifically, clinical trials assessing the efficacy of full, regular doses of psychedelics on treatment resistant depression (28) and end of life depression and anxiety (13, 29, 30) have found an association between the acute quality of the experience (including occurrence of profound psychological ‘peak’ or ‘mystical’ experiences), and long-term (positive) clinical outcomes. It could thus be suggested that the acute psychedelic experience is a valued or even necessary aspect of psychedelic-assisted therapy in treating depression and anxiety disorders, and would help explain why doses too low to induce a noticeable change in consciousness would be rated as less effective. Furthermore, as a dose-specific difference in SRE of neurodevelopmental and physiological disorders was not seen, it could be hypothesized that such an experience is not necessary for these disorders, suggesting a different mode of therapeutic action. However, future clinical studies need to properly assess this, as well as further explore whether effects are specific and not due to other currently unmeasured components of psychedelic therapy (28), and investigate the neurobiological mechanisms underlying the acute quality of the experience.

This study is not without its limitations. As our population of interest were recreational psychedelic users, it might not be a representative sample in terms of prevalence of mental disorders. However, data shows that these rates were in line with the general population worldwide (31), with most frequently diagnosed disorders in our sample being stress-related disorders, i.e., depression (*N* = 299; 72.9%) and anxiety (*N* = 228; 55.6%). Furthermore, complex mental comorbidity was the rule rather than the exception while the majority (72.7%) of our sample indicated to be diagnosed with more than one disorder, which is also the case in the ‘general’ psychiatric population (32).

Additionally, comparison of effectiveness of different kind of psychedelics was not possible due to the low cell count for some of the separate substances. Future studies might focus on the effectiveness of LSD compared to psilocybin, for example, as anecdotal reports state that microdosing with LSD produces more stimulating effects compared to psilocybin (11). LSD could therefore be less suited in the treatment of anxiety disorders, as anxiety is already a state of hyperarousal (33), and more suitable in disorders characterized by biological hypo-arousal which is the case in ADHD (34). In addition, the sample was too small in order to make a comparison between microdosing and the different kind of offered treatments, such as medication or therapy sessions.

Moreover, disorder history (duration and severity) were not assessed, so it cannot be established whether microdosing was rated to be more effective for more or less severe cases. Additionally, the duration of symptom alleviation was not asked, it might be that conventional treatment only lasts for one day, microdosing might only last for a couple of days, while regular doses might relieve symptoms up to several months. Finally, as the survey was presented on psychedelic fora, the self-selected sample might have been biased towards the favorability of psychedelics over all kinds of treatments. Thus, the results should be interpreted with caution, and used for rationale to further assess indications of therapeutic potential of psychedelic substances.

To conclude, this study demonstrates that SRE of MDP to alleviate symptoms of a range of mental or physiological diagnoses is higher compared to conventionally offered treatment options and lower than regular (‘full’) psychedelic doses. Future RCTs in patient populations will be able to answer questions of these effectivity claims of psychedelics, whether these are dose related, disorder specific, and superior to conventional treatments.

## Data Availability

The datasets generated for this study are available on request to the corresponding author.

## Ethics Statement

The studies involving human participants were reviewed and approved by Ethics Review Committee of Psychology and Neuroscience, Maastricht University, Maastricht, the Netherlands. The patients/participants provided their written informed consent to participate in this study.

## Author Contributions

KK, NM, and PD designed the study. NM collected the data. NH analyzed the data. NH, NM, and KK wrote the manuscript. All authors contributed to manuscript revision, read, and approved the submitted version.

## Conflict of Interest Statement

The authors declare that the research was conducted in the absence of any commercial or financial relationships that could be construed as a potential conflict of interest.
